# Use of Trial Register Information during the Peer Review Process

**DOI:** 10.1371/journal.pone.0059910

**Published:** 2013-04-10

**Authors:** Sylvain Mathieu, An-Wen Chan, Philippe Ravaud

**Affiliations:** 1 INSERM U738, Centre d'épidémiologie Clinique, French Cochrane Center, University Paris Descartes et Hotel Dieu, Paris, France; 2 Department of Rheumatology, University Clermont 1, Clermont-Ferrand, France; 3 Women's College Research Institute, University of Toronto, Toronto, Ontario, Canada; University of Illinois-Chicago, United States of America

## Abstract

**Introduction:**

Evidence in the medical literature suggests that trial registration may not be preventing selective reporting of results. We wondered about the place of such information in the peer-review process.

**Method:**

We asked 1,503 corresponding authors of clinical trials and 1,733 reviewers to complete an online survey soliciting their views on the use of trial registry information during the peer-review process.

**Results:**

1,136 authors (n = 713) and reviewers (n = 423) responded (37.5%); 676 (59.5%) had reviewed an article reporting a clinical trial in the past 2 years. Among these, 232 (34.3%) examined information registered on a trial registry. If one or more items (primary outcome, eligibility criteria, etc.) differed between the registry record and the manuscript, 206 (88.8%) mentioned the discrepancy in their review comments, 46 (19.8%) advised editors not to accept the manuscript, and 8 did nothing. The reviewers' reasons for not using the trial registry information included a lack of registration number in the manuscript (n = 132; 34.2%), lack of time (n = 128; 33.2%), lack of usefulness of registered information for peer review (n = 100; 25.9%), lack of awareness about registries (n = 54; 14%), and excessive complexity of the process (n = 39; 10.1%).

**Conclusion:**

This survey revealed that only one-third of the peer reviewers surveyed examined registered trial information and reported any discrepancies to journal editors.

## Introduction

Several authors have shown evidence of selective outcome reporting in published results of randomized controlled trials (RCTs) [1]–[3]. In 2004, Chan *et al*. reported a difference in primary outcomes between trial protocols and publications for 40% of the trials analysed^2^. Another study found that for 62% of trial publications, at least one primary outcome was changed, introduced, or omitted as compared with the submitted protocol [1]. Statistically significant outcomes are about three times more likely to be reported in a publication than non-significant outcomes. Outcome reporting bias is widely accepted as a major problem deserving more attention.

In 2005, the International Committee of Medical Journal Editors (ICMJE) initiated a policy requiring investigators to record basic information about their RCT into a clinical trial registry before participant enrollment as a pre-condition for publishing the trial's findings [4]. A major goal of trial registration is to enhance transparency and accountability in the planning, execution, and reporting of clinical trials [4]–[6]. Registration should help to identify and deter the biased suppression of trials and results. Although trial registration is a major step toward reducing reporting biases, many trials remain unregistered and the quality and timing of registration still need improvement [7]–[19].

Previous studies have shown that selective outcome reporting remains prevalent among registered trials [20]–[22]. In a recent survey, we analyzed reports for 323 RCTs and compared them to their respective registration records and found that only 147 studies (45.5%) were correctly registered (*i.e.,* registered before the study ended with a clearly defined primary outcome). Among articles for adequately registered trials, 31% (46/147) showed discrepancies between the registered and published outcomes. These data suggest that registered information is not sufficiently being consulted to identify unacknowledged changes to primary outcomes during the manuscript review process.

We surveyed the journal reviewers and corresponding authors of recently published reports of RCTs to determine their views on the use of trial registries during the peer-review process.

## Methods

### Ethics statement

The present research is not considered as biomedical research according to the French legislation [23]. Therefore, no ethical approval was required according to the related statement of our referent research ethics committee (Comité de Protection des Personnes Sud-Est VI, Clermont-Ferrand, France; chair: Pr. J.E. Bazin). All data has been analysed anonymously.

### Participant inclusion

We included reviewers of manuscripts of RCTs, including corresponding authors and journal reviewers. To identify the corresponding author of RCT articles recently indexed in PubMed, we used the search term “randomized controlled trial” on 19/12/2011 to identify reports of RCTs published in 2010 and 2011. We also applied the highly sensitive search strategy described by Robinson and Dickersin [24]. Our search focused on human studies without any limitation by medical area, language, or type of intervention. Titles, names of the corresponding author, and abstracts were screened by one author (SM). The e-mail addresses of corresponding authors were checked first in the authors' affiliations on PubMed, and if that yielded no results, the full-text article was checked for an e-mail address. We also looked for e-mail addresses on the Internet using Google. If no e-mail address for the author was found or if we were not sure that a given e-mail address corresponded to the corresponding author, the article was excluded from the analysis.

We obtained the list of reviewers of *New England Journal of Medicine* (*NEJM*), *Journal of the American Medical Association* (*JAMA*), and *Annals of the Rheumatic Diseases* (*ARD*) from the last journal issues for the years 2010 or 2011, which thank reviewers for their contributions. The e-mail address search was the same as for corresponding authors, namely, first a PubMed search, then a search on the Internet via Google. We included all reviewers of these three journals, whether they reviewed clinical trials or not.

If a corresponding author or reviewer appeared more than once in the participant search, only the most recent e-mail address was retrieved. From April to May 2012, the participants were sent e-mails inviting them to complete an online survey (Appendices S2 and S3), explaining that participation was voluntary, and that their identities and responses would be kept confidential. Up to two reminder emails were sent to non-responders at about one-week intervals. No incentive or compensation was offered for participation.

### Survey instrument

The survey asked participants about their knowledge, current practices, and opinions regarding trial registration and peer review of manuscripts (Appendix S1). The questionnaire was pilot-tested on a convenience sample of trial investigators and methodologists.

We recorded the peer reviewers' research affiliations, along with the number of annual peer reviews they completed and the number of trials they had joined as investigators. The survey comprised two parts: 1) the reviewer's experiences based on the most recent manuscript reviewed over the previous two years, and 2) whether they compared registered information with manuscript information during the review process, and if so, whether they reported any discrepancies. For participants who responded that the reviewer should verify trial registration or check for discrepancies, we asked them to rate the effectiveness of various methods to facilitate the comparison of registered and reported information during the peer-review process. Ratings of 4 and 5 on a scale from 1 to 5 were considered effective (Appendix S1).

### Statistical analysis

Web-based surveys produce variable response rates [25], [26]. We estimated the *a priori* response rate to be about 30%, assuming that 5% of e-mails would not reach their intended recipient and that some trialists would not have reviewed an article within the previous two years. We sought about 500 survey responses to ensure a sufficient sample size. Data are represented descriptively as proportions. Categorical variables were analyzed by chi-square test. Statistical analysis involved use of R software (http://www.R-project.org, the R Foundation for Statistical Computing, Vienna, Austria).

## Results

### Response to the survey

We surveyed 1,503 corresponding authors of recently published RCTs indexed in PubMed, and 1,777 peer reviewers for *NEJM* (n = 227), *JAMA* (n = 773), and *ARD* (n = 777). The flow of participants is in Figure S1. Of the 3,033 participants invited to complete the survey, 1,165 responded and 1,136 respondents submitted useable data (37.5%): 713 from authors and 423 from reviewers.

### Respondent characteristics

Of the 1,136 respondents, most (n = 931; 82%) were affiliated with a university hospital (Table S1). Only 11 worked in the pharmaceutical industry, and 43 worked in government. In all, 41% (n = 467) had been investigators for 1 to 5 trials, 18.0% (n = 204) had been investigators for more than 20 trials. Between 2007 and 2011, half of the respondents had reviewed 1 to 10 articles. Only 51 and 67 reviewed 0 and more than 50 articles, respectively; 60% (n = 676) had reviewed an article in the previous two years. Four respondents that belonged to the journal list of reviewers had reviewed no article since 2007.

The percentage of trial investigators and the average number of published articles reviewed per year from 2007 to 2011 were higher in the reviewer group than in the author group (Table S1).

### Experience based on the most recently reviewed trial manuscript

Of the 676 respondents who had peer reviewed an article in the previous two years, 58 responses were missing or not useable for analysis. Overall, 232 respondents (34.3%) had examined the information registered on a trial registry; most were corresponding authors (n = 154; 66.4%) and 78 were journal reviewers. The most commonly reviewed items in the registration record were the primary outcome (94.4%), eligibility criteria (83.2%), planned sample size (83.2%), secondary outcomes (81%), and posted results (47.8%). When one or more of the above methodological components differed between the registry record and the manuscript, 206 respondents (88.8%) mentioned the discrepancy in their review comments, 46 (19.8%) advised editors not to accept the reported manuscript, and 8 did nothing. Four respondents indicated that their recommendations to journal editors differed depending on the importance of the discrepancies.

For 386 respondents who did not look at registry information during the peer review, the most common reasons included a lack of registration number in the manuscript (n = 132, 34.2%), lack of time (n = 128, 33%), lack of useful registered information (n = 100, 26%), lack of awareness of the availability of registered information (n = 54, 14%), excessive complexity of the process (n = 39, 10.1%), lack of registration record (n = 34, 8.3%) and not remembering to check the registry record (n = 26, 6.7%). Overall, 13 participants reported not having looked at the trial registry because they thought that the journal had done so before sending the manuscript to review.

### Proposals for facilitating the use of registered information during peer review

For the 676 participants who had peer reviewed an article in the previous two years, 317 (46.9%) and 182 (26.9%) felt that the managing or academic editor, respectively, was responsible for checking that the trial was registered. Only 50 respondents (7.4%) believed that the peer reviewer should have this responsibility. Other proposed responsible parties were the editor-in-chief (n = 21), investigator (n = 13), research ethics committee (n = 4), and sponsor (n = 3). Ten participants thought that it was not useful to check whether a trial was registered. By contrast, respondents considered a more evenly divided responsibility for checking for consistency between the registered information and the manuscript (academic editor, n = 210 [31.1%]; managing editor, n = 177 [26.2%]; peer reviewer, n = 170 [25.1%]).

The 177 respondents who felt that the peer reviewer should verify that a trial was registered or discrepant with the manuscript were asked to rate the effectiveness of certain options for facilitating the peer reviewer's ability to compare registries and manuscripts. The three most effective options were providing peer reviewers with a direct Web link to the corresponding trial record on the registry website (n = 112; 63.3%), providing a list of the registered information to accompany the full manuscript (n = 90; 50.8%), and providing the registration number with the manuscript (n = 80; 45.2%).

## Discussion

We surveyed corresponding authors and peer reviewers of medical journals about the role of registered trial information in the peer-review process. Of the 676 respondents who had peer reviewed a manuscript reporting the results of a clinical trial in the past two years, only one-third examined the registered information for the trial. When discrepancies were identified, most respondents (88.8%) mentioned them in their review comments, and 19.8% advised editors not to accept the manuscript.

Despite measures to improve trial transparency and prevent selective outcome reporting, recent studies reveal that these issues remain prevalent but are identifiable using registered trial information [14], [16], [17]. We found that two-thirds of respondents reviewing articles did not look at trial registry information for several reasons, mostly related to difficulty or inconvenience in accessing the registry record. Our respondents agreed that specific actions could facilitate their use of registered information. A possible improvement in the peer-review process could be that journals routinely provide peer reviewers with the trial registration number and a direct Web link to the corresponding registry record, or provide the registered information with the manuscript. In keeping with ICMJE policy, manuscripts that are submitted to journals should be registered and could contain a trial registration number. The ICMJE policy recommends that “The ICMJE member journals will require, as a condition of consideration for publication in their journals, registration in a public trials registry. ICMJE journals will consider [reports of] trials beginning on or after July 1, 2005 only if registration occurred before the first patient was enrolled (‘prospective registration’)”.

Alternatively, as several of our respondents suggested, managing or academic editors could themselves verify that the trial had been registered before patient recruitment and could check that there are no discrepancies between the registered and published items (primary outcome, inclusion and exclusion criteria, sample size, time of assessment, etc.) before sending the manuscript to peer reviewers.

One-quarter of respondents who did not examine the registry record when they peer reviewed a manuscript stated that the reason was a perceived lack of usefulness of registered information. Previous studies have found that the completeness and quality of information in registries is variable. Zarin et al. assessed ClinicalTrials.gov data that were publicly available for a sample of 3,284 registered trials. Of the 2,324 posted results entries that had been posted publicly, 14% were linked to a PubMed citation through an indexed ClinicalTrials.gov registration number. Of 2,178 clinical trials with posted results records, 20% had more than two reported primary outcomes measures and 5% more than five. For some studies, posted results include more than 100 primary and secondary outcome measures [16]. The medical community should emphasize the importance of trial registration and ensure that proper information is submitted to registries. There may be legitimate discrepancies between the manuscript and registry record, but these should be transparently reported in the paper or in the registry so that readers can judge the potential for bias. When incomplete or uninformative information is provided in registry records, journals could consider these trials inadequately registered and could ask authors to complete this information or explain why the study is not correctly registered. The objective of this article is not to state that an article must be rejected by editors or reviewers in case of discrepancy but that 1) it is necessary to verify whether the published paper is in agreement with is the registered information (and if there are changes over time, whether it is theoretically possible and mandatory to register them) and 2) in case of discrepancies, it is necessary and theoretically possible to state the reason for that.

According to previous studies, using the Internet to conduct surveys is associated with a variable response rate (from 9% to 94%) and a lower rate than with a traditional mailed survey [25], [27]. We obtained a moderate response rate of 37.5%.

This study has several limitations. Our sample of authors who published trials in PubMed-indexed journals should be broadly generalizable, but we also surveyed a sample of reviewers of medical journals with high impact factors, which could differ systematically from reviewers of other journals. Indeed, these reviewers may have been more aware of the issues of trial registration and discrepancies between the registered protocol and the published results. Our results may thus overestimate the use of trial registration information by peer reviewers. Another limitation might be that we provided the respondents with a list of pre-defined options for improving the peer-review process. However, we added an open-question to solicit any other suggestions.

We reveal that only one-third of peer reviewers of articles of RCTs use the information recorded on the trial registry website, and most report any discrepancies to journal editors. The scientific and medical communities could emphasize the value and role of trial registration to facilitate peer review of trial manuscripts and could introduce concrete measures to facilitate the access to and use of trial registry information.

## Supporting Information

Figure S1 **Flow chart of participants in the study.**
(TIF)

Table S1 Respondent characteristics.
(DOCX)

Appendix S1 **Web questionnaire.**
(DOCX)

Appendix S2 **Mail for corresponding authors.**
(DOCX)

Appendix S3 **Mail for reviewers.**
(DOCX)
