# Supplementary material for: Use of Trial Register Information during the Peer Review Process
Source: PLoS One. 2013 Apr 10;8(4):e59910. doi: 10.1371/journal.pone.0059910 (PMC3622662; doi:10.1371/journal.pone.0059910)
Supplement: Table S1 — Respondent characteristics. (DOCX) [file pone.0059910.s002.docx]

**Table 1. Respondent characteristics**

|  | All  (n=1,136) | Authors  (n=713) | Reviewers  (n=423) |
| --- | --- | --- | --- |
| **Research affiliation** |  |  |  |
| University hospital and department | 931 (82.0) | 571 (80.1) | 360 (85.1) |
| Community hospital or clinic | 38 (3.3) | 24 (3.4) | 14 (3.3) |
| Industry | 11 (1.0) | 11 (1.5) | 0 (0.0) |
| Government  Private non-profit | 43 (3.8)  43 (3.8) | 30 (4.2)  27 (3.8) | 13 (3.1)  16 (3.8) |
| Other  Unknown | 38 (3.3)  32 (2.8) | 27 (3.8)  23 (3.2) | 11 (2.6)  9 (2.1) |
| No. **of trials with a role of investigator** |  |  |  |
| 1-5  6-10 | 467 (41.1)  237 (20.9) | 327 (45.9)  142 (19.9) | 140 (33.1)  95 (22.5) |
| 11-15 | 98 (8.6) | 47 (6.6) | 51 (12.1) |
| 16-20 | 77 (6.8) | 43 (6.0) | 34 (8.0) |
| > 20  Unknown | 204 (18.0)  53 (4.7) | 120 (16.8)  34 (4.8) | 84 (19.9)  19 (4.5) |
| **Mean no. of published articles reviewed each year from 2007-2011** |  |  |  |
| 0  1-5  6-10  11-20  21-50  >50  Unknown | 67 (5.9)  317 (27.9)  280 (24.6)  223 (19.6)  131 (11.5)  67 (5.9)  51 (4.5) | 63 (8.8)  253 (34.5)  151 (21.2)  115 (16.1)  61 (8.6)  28 (3.9)  42 (5.9) | 4 (0.9)  64 (15.1)  129 (30.5)  108 (25.5)  70 (16.5)  39 (9.2)  9 (2.1) |

Data are number (%)
