# Supplementary material for: Use of Trial Register Information during the Peer Review Process
Source: PLoS One. 2013 Apr 10;8(4):e59910. doi: 10.1371/journal.pone.0059910 (PMC3622662; doi:10.1371/journal.pone.0059910)
Supplement: Appendix S1 — Web questionnaire. (DOCX) [file pone.0059910.s003.docx]

**APPENDIX 1. WEB QUESTIONNAIRE**

**Your personal characteristics as a journal peer reviewer**

Your research affiliation (Select the single best answer)

University Hospital or Department Community Hospital or Clinic

Government  Private nonprofit (e.g., ___)

Industry  Other (please specify) ___________________

The total number of completed trials in which you have ever participated as an investigator:

1-5 6-10  11-15 16-20 >20

The average number of published articles you have reviewed **per year** from 2007-2011:

0 1-5 6-10  11-20  21-50 >50

Are you a native English-speaker? Yes  No

In the past two years, have you peer-reviewed a journal manuscript reporting the main results of a clinical trial? Yes  No

**Based on the most recent trial manuscript you peer-reviewed over the past 2 years:**

1) Did you review the information registered on a trial registry (such as ClinicalTrials.gov) for the particular trial?  Yes  No

**If NO:**

- Why?(Several responses possible)

No registration number stated in the manuscript

No registration record was found

Registered information is generally not useful for the peer review process

Not aware that this information is available on registries

Lack of time

Too complicated

It is not important to check registered information during the peer review process

Other reason(s):___________________

**If YES:**

- Did you check the:

- Definition of primary outcomes (specific variable, timing of assessment)?

Yes  No Unable to respond (UR)

- Definition of secondary outcomes?  Yes  No UR

- Eligibility criteria?  Yes  No UR

- Planned sample size?  Yes  No UR

- Posted results? Yes No Not available on the particular registry

UR

- Other (please specify)

- What was or what would be your reaction if one or more of the above methodological components or results differed between the registry record and manuscript? (Check all that apply)

Mention the discrepancy in your peer-review comments Advise editors not to accept the reported manuscript

Nothing

Other (Please specify)___________________

**Facilitating the comparison of the registered and reported information during the peer-review process**

1) Who do you believe should verify that the trial was registered prospectively prior to recruiting participants?

Peer reviewer Academic editor Managing editor Other(s) (please specify)___________________

2) Who do you believe should check for any discrepancies between the registered information and the manuscript?

Peer reviewer Academic editor Managing editor Other(s) (please specify)___________________

On a scale of 1 to 5 (5 = most effective; 1 = least effective), please rate the effectiveness of the following options for facilitating the peer reviewer’s comparison of registered information with corresponding information from the manuscript:

- Providing the trial registration number with the manuscript to review

1  2  3  4  5

-Providing the reviewer with a direct Web link to the corresponding protocol on the registration website  1  2  3  4  5

- Providing the reviewer with a list of registered information to accompany the full manuscript  1  2  3  4  5

- Providing the reviewer with a list of the registered items, along with manuscript page numbers where corresponding information could be found. 1  2  3  4  5

- Providing the reviewer with a list of the registered items, along with corresponding text quoted from the manuscript for each registered item  1  2  3  4  5

-Please provide any other proposition(s) to facilitate the article reviewing:
