# Supplementary material for: Use of Trial Register Information during the Peer Review Process
Source: PLoS One. 2013 Apr 10;8(4):e59910. doi: 10.1371/journal.pone.0059910 (PMC3622662; doi:10.1371/journal.pone.0059910)
Supplement: Appendix S3 — Mail for reviewers. (DOCX) [file pone.0059910.s005.docx]

**APPENDIX 3. MAIL FOR REVIEWERS**

Dear colleague,

We are conducting a survey about clinical trial registration and the journal peer review process. You have been included in our sample based on your role as a peer reviewer in high impact factor journal.

The registration of clinical trials on public registries *(e.g.,* ClinicalTrials.gov, ISRCTN, etc.) has become an important responsibility in medical research. Despite these efforts towards improved transparency, several reviews have found persistent selective outcome reporting when registered information was compared to journal publications.

Changes to the journal peer review process could help reduce this reporting bias. We would greatly appreciate your views on clinical trial registration based on your role as a journal peer reviewer. This participation would require about 5 minutes of your time and would be done via an **online Web-based Survey**. Your answers will be anonymous and treated confidentially.

Thank you in advance for your participation.

Sincerely,

Sylvain MATHIEU (1,2), An-Wen CHAN (3), and Philippe RAVAUD (1)

(1) Centre of Clinical Epidemiology, INSERM U738; Paris, France; University Paris Descartes
(2) Department of rheumatology, Clermont-Ferrand, France; University Clermont 1
(3) Women's College Research Institute, University of Toronto, Toronto, Ontario, Canada.


Pr Philippe Ravaud
Centre d'épidémiologie Clinique
French Cochrane center
HôtelDieu,Université Paris Descartes
Phone: +33 (0) 1 42 34 89 86 ,sec 01 42 34 89 87
Fax: +33 (0) 1 40 25 67 73
[philippe.ravaud@htd.aphp.fr](https://owa.chu-clermontferrand.fr/owa/redir.aspx?C=f9a1be3b6fdb4916ba861d309f45b329&URL=mailto%3Aphilippe.ravaud%40htd.aphp.fr)
